# Diabetes mellitus mortality in a municipality in the state of São Paulo, 2010 to 2014

**DOI:** 10.11606/S1518-8787.2019053000561

**Published:** 2019-02-20

**Authors:** Rafael Aparecido Dias Lima, Plinio Tadeu Istilli, Carla Regina de Souza Teixeira, Maria Lúcia Zanetti, Maria Tereza da Costa Gonçalves Torquato

**Affiliations:** I Universidade de São Paulo. Escola de Enfermagem de Ribeirão Preto. Programa de Graduação em Enfermagem. Ribeirão Preto, SP, Brasil; II Universidade de São Paulo. Escola de Enfermagem de Ribeirão Preto. Programa de Pós-Graduação em Enfermagem Fundamental. Ribeirão Preto, SP, Brasil; III Prefeitura de Ribeirão Preto. Secretaria Municipal da Saúde de Ribeirão Preto. Programa de Atenção às Pessoas com Doenças Crônicas não Transmissíveis. Ribeirão Preto, SP, Brasil

**Keywords:** Diabetes Mellitus, mortality, Risk Factors, Time Series Studies, Diabetes Mellitus, mortalidade, Fatores de Risco, Estudos de Séries Temporais

## Abstract

**OBJECTIVE:**

To describe diabetes mellitus mortality according to sex and age in a municipality in the state of São Paulo, in the period ranging from 2010 to 2014.

**METHODS:**

This was a temporal series ecological study carried out in Ribeirão Preto, state of São Paulo. The data was comprised of information on 583 deaths of Ribeirão Preto residents – regardless of the place of death – from 2010 to 2014. The data source was the electronic system of the Epidemiological Surveillance of the Municipal Health Department of the evaluated municipality. Sex, age group, premature death and year of death were chosen as variables. Subsequently, age-standardized mortality rates were calculated using the World Health Organization's standard population, in addition to total and average per death potential years of life lost.

**RESULTS:**

Mortality due to diabetes mellitus in the municipality increased during the studied period. There was a higher occurrence of female deaths, especially in the ≥ 80 years age group. The highest rates of age-standardized mortality were male. For both sexes, there was an annual mean increase of 9% in premature mortality during the studied period. Diabetes decreased life expectancy by 10 years.

**CONCLUSIONS:**

As a diagnosis of local health care, the significant increase in age-standardized mortality rates, premature mortality and potential years of life lost in the studied municipality point to the need for improvements in health promotion and disease prevention measures. It is our hope that the results presented in this study contribute to the monitoring of mortality rates in the coming years.

## RESUMO

**OBJETIVO:**

Descrever a mortalidade por diabetes *mellitus* segundo sexo e idade em um município do estado de São Paulo, 2010–2014.

**MÉTODOS:**

Estudo ecológico, de série temporal, realizado em Ribeirão Preto, SP. Foram investigados 583 óbitos de pessoas residentes no município independentemente do local de óbito, no período de 2010 a 2014. A fonte de dados foi o sistema eletrônico da Vigilância Epidemiológica da Secretaria Municipal de Saúde do município avaliado. Elegeram-se as variáveis sexo, faixa etária, morte prematura e ano de ocorrência do óbito. Posteriormente, foram calculadas as taxas de mortalidade padronizada por idade, utilizando a população padrão da Organização Mundial de Saúde, além do total e média por óbito dos anos potenciais de vida perdidos.

**RESULTADOS:**

A mortalidade por diabetes *mellitus* no município aumentou no período estudado. Houve maior ocorrência dos óbitos no sexo feminino, principalmente na faixa etária ≥ 80 anos. Os maiores índices da taxa de mortalidade padronizada por idade foram no sexo masculino. Em ambos os sexos, houve aumento médio anual de 9% na mortalidade prematura no período estudado. O diabetes diminui 10 anos da expectativa de vida.

**CONCLUSÕES:**

O aumento expressivo das taxas de mortalidade padronizada por idade, de mortalidade prematura e dos anos potenciais de vida perdidos no município do estudo entre 2010 a 2014 viabilizaram diagnóstico local de saúde com necessidades de melhorias nas medidas de prevenção e promoção da saúde. Espera-se que os resultados apresentados neste estudo contribuam para o monitoramento dos índices de mortalidade nos próximos anos.

## INTRODUCTION

Due to being associated with high mortality rates, cardiovascular diseases, neoplasms, chronic respiratory diseases and diabetes mellitus (DM) are considered the main chronic noncommunicable diseases (CNCDs). The World Health Organization (WHO) has estimated a total of 38 million deaths from CNCDs in 2012, corresponding to 70% of all worldwide deaths^1^.

Among the four major CNCDs in 2012, cardiovascular diseases caused 17.5 million deaths, followed by neoplasms (8.2 million), chronic respiratory diseases (4 million) and DM (1.5 million). Furthermore, 16 million (42%) CNCD deaths were premature and preventable^1^.

DM in particular has been increasing worldwide. The number of people with DM increased from 108 million in 1980 to 422 million in 2014^2^. In 2015, about five million people aged 20–79 died from DM, more than the sum of infectious-disease deaths in 2013^3^ (1.5 million deaths from HIV/AIDS, 1.5 million deaths from tuberculosis, and 0.6 million deaths from malaria). A study showed that, from 1980 to 2012, 955,455 people aged 20 years or more died from DM in Brazil. The same study also showed that when considering DM as the underlying or associated cause of mortality, the number of deaths increased to 1,076,434, reiterating how monitoring the DM population is important for the health system^4^.

Among the policy strategies to reduce the burden of DM mortality are the Global Action Plan for the Prevention and Control of NCDs, by the World Health Organization (WHO)^5^, and the Strategic Action Plan to Tackle Noncommunicable Diseases in Brazil, 2011–2022, by Brazil's Ministry of Health. The latter has three pillars: surveillance, health promotion, and comprehensive care^6^.

In the surveillance pillar, a mortality indicator aggregates and organizes information on population gaps related to morbidity, mortality and their risk factors. Information, evaluation and monitoring aim to define the population's vulnerability, and subsequently contribute to the qualification of health interventions, as well as the reduction of mortality rates^7^. Monitoring should be performed at all system management levels, from the municipal to the national^8^.

In view of the above, this study aims to describe DM mortality, according to sex and age, in a municipality in the state of São Paulo, from 2010 to 2014. It is our hope that this study is able to provide subsidies for DM monitoring, a fundamental activity of the Health Surveillance System, and contribute to the development of effective strategies in diabetes education.

## METHODS

This was an ecological and time-trend study^9^, carried out in the city of Ribeirão Preto, located in the northeastern region of the state of São Paulo, 313 kilometers from its capital. The municipality has a total area of 650 km^2^ and a population of 604,682 inhabitants, according to the 2010 census^10^.

The study population was comprised of deaths of residents of the municipality, regardless of the place of death, taking place from 2010 to 2014. These data were obtained from the Epidemiological Surveillance Division of the Municipal Health Department of Ribeirão Preto, state of São Pauilo, in 2017, after approval by the Ethics Committee of the School of Nursing of the University of São Paulo at Ribeirão Preto (EERP-USP), Process 2,111,771. Among 718 DM deaths, 135 were excluded, as the individuals were not residents of the studied municipality.

The data were segregated by the following variables: sex (female and male), age (in years) classified by age group (0–4; 5–9; 10–14; 15–19; 20–24; 25–29; 30–34; 35–39; 40–44; 45–49; 50–54; 55–59; 60–64; 65–69; 70–74; 75–79; ≥ 80 years), date of death, and cause of death. Age ranges were classified according to the Tab for Windows program (TABWIN), a free piece of software developed by Datasus^11^. The basic cause of death per the death certificate, and DM cases classified as E10 to E14 (according to the WHO's ICD-10 – International Classification of Diseases and Related Health Problems, 10th revision), were included in this study. E10 refers to insulin-dependent DM; E11, to non-insulin-dependent DM; E12, to malnutrition-related DM; E13, to other specified types of DM; and E14, to unspecified DM^1^ ^–^ ^12^.

The obtained data were imported into Excel spreadsheets, for descriptive treatment and subsequent statistical analysis. In the first stage of analysis, deaths of Ribeirão Preto, residents were selected for inclusion, based on the deceased's documented city code. Statistical descriptive data analysis was performed using the Statistical Program For Social Sciences (SPSS), version 22, for Windows. Descriptive data was presented using frequency and percentage of DM deaths spanning the 2010 to 2014 period, according to year, sex and age group.

DM age-standardized mortality rates (ASMR) per 100,000 inhabitants were calculated according to gender, age group and year of death, using WHO's standard population and the direct standardization method^13^ ^,^ ^14^. For individuals with 80 years of age or above, the number of deaths and the percentages established by the WHO were added together.

For premature mortality (people with ≥ 30 and ≤ 69 years of age), the methodology proposed by the WHO was employed^6^. Potential years of life lost (PYLL) were estimated using the method proposed by Romeder and McWhinnie^15^.

In order to gauge the period's rate reduction percentages, the annual rate reduction was initially calculated by dividing the difference between consecutive year rates by the rate in the initial year of the calculation (multiplied by 100). The resulting mean was defined as the period's annual reduction^14^.

## RESULTS

In 2010–2014, a total of 583 deaths were attributed to DM in Ribeirão Preto, state of São Paulo. The DM type was unspecified for 494 (84.7%) deaths. Acute complications (final character of the ICD-10 code: .0 or .1) were attributed to 10 (1.7%) deaths. As for complication types, 161 (34.5%) deaths were due to renal complications, 68 (11.7%) due to peripheral circulatory complications, 52 (8.9%) due to multiple complications, and 24 (4.1%) due to other complications. It should be noted that 228 (39.1%) deaths had no record of complications (Table 1).

**Table 1:** Absolute number (n) and percentage (%) distribution of deaths by diabetes mellitus, according to ICD-10 classification, Ribeirão Preto, 2010–2014. Ribeirão Preto, state of São Paulo, Brazil, 2017.

ICD-10 final character	ICD-10 code					Total	
	E10^a^	E11^b^	E12^c^	E13^d^	E14^e^		
	Number of deaths					n	%
Comatose	0	3	0	0	1	4	0.7
.1 Ketoacidosis	0	0	0	0	6	6	1.0
.2 Renal complications	6	34	0	0	161	201	34.5
.5 Peripheral circulatory complications	0	7	0	0	61	68	11.7
.6 Other specified complications	0	0	1	0	0	1	0.2
.7 Multiple complications	1	6	1	0	44	52	8.9
.8 Unspecified complications	0	1	0	0	22	23	3.9
.9 No complications	5	24	0	0	199	228	39.1
N total	12	75	2	0	494	583	100
%	2.1	12.9	0.3	0	84.7	100	

Source: Epidemiological Surveillance Division of the Municipal Secretariat of Ribeirão Preto.

a E10 – insulin-dependent diabetes mellitus.

b E11 – non-insulin-dependent diabetes mellitus.

c E12 – malnutrition-related diabetes mellitus.

d E13 – other specified types of diabetes mellitus.

e E14 – unspecified diabetes mellitus.

Most deaths – 321 (55.1%) – were female. The predominant age group was older people over 80 years of age (n = 194, 33.3%). There was an expressive increase in the total number of deaths from 55 years of age onward (Table 2).

**Table 2:** Absolute number (n), percentage (%) and age-standardized mortality rate (ASMR) distribution for diabetes mellitus deaths of Ribeirão Preto residents, 2010 to 2014, according to sex and age group. Ribeirão Preto, state of São Paulo, Brazil, 2017.

Age group	Male sex			Female sex			Total		
	n	%	ASMR (per 100,000 inhabitants)	n	%	ASMR (per 100,000 inhabitants)	n	%	ASMR (per 100,000 inhabitants)
0–4	0	0	0.0	0	0	0.0	0	0	0.0
5–9	1	0.2	0,4	0	0	0.0	1	0.2	0.2
10–14	0	0	0.0	0	0	0.0	0	0	0.0
15–19	0	0	0.0	1	0.2	0.4	1	0.2	0.2
20–24	1	0.2	0.3	1	0.2	0.3	2	0.3	0.3
25–29	1	0.2	0.3	1	0.2	0.3	2	0.3	0.3
30–34	2	0.3	0.6	0	0	0.0	2	0.3	0.3
35–39	5	0.9	1.6	1	0.2	0.3	6	1.0	1.0
40–44	4	0.7	1.3	8	1.4	2.4	12	2.1	1.9
45–49	4	0.7	1.3	1	0.2	0.3	5	0.9	0.7
50–54	10	1.7	3.2	9	1.5	2.4	19	3.3	2.8
55–59	30	5.2	9.8	14	2.4	3.8	44	7.6	6.6
60–64	31	5.3	10.9	35	6.0	9.8	66	11.3	10.3
65–69	43	7.4	16.9	32	5.5	9.6	75	12.9	12.8
70–74	34	5.8	13.3	31	5.3	8.5	65	11.2	10.5
75–79	34	5.8	13.3	55	9.4	13.9	89	15.3	13.7
80+	62	10.6	24.8	132	22.7	26.9	194	33.3	26.2
Total	262	44.9	90.3	321	55.1	102.1	583	100	96.4

Source: Epidemiological Surveillance Division of the Municipal Secretariat of Ribeirão Preto.

Regarding sex, women had a mean of 64.2 deaths/year, and men had a mean of 52.4 deaths/year. Regarding ASMR, there was a 38.5% increase from the first to the last year of the study, in both sexes (Table 3).

**Table 3:** Absolute number (n), percentage (%), and age-standardized mortality rate (ASMR) distribution for diabetes mellitus deaths of Ribeirão Preto residents, 2010 to 2014, according to sex and year. Ribeirão Preto, state of São Paulo, Brazil, 2017.

Year	Male sex			Female sex			Total		
	n	%	ASMR (per 100,000 inhabitants)	n	%	ASMR (per 100,000 inhabitants)	n	%	ASMR (per 100,000 inhabitants)
2010	47	8.1	17.5	57	9.8	14.1	104	17.8	15.6
2011	51	8.7	19.2	46	7.9	11.5	97	16.6	14.7
2012	54	9.3	20.3	69	11,8	17.0	123	21.1	18.5
2013	51	8.7	19.1	63	10.8	15.4	114	19.6	17.2
2014	59	10.2	21.8	86	14.7	20.9	145	24.9	21.6

Source: Epidemiological Surveillance Division of the Municipal Secretariat of Ribeirão Preto.

Among 583 deaths, 229 (39.3%) were considered premature. Among premature deaths, 129 (22.1%) were male. The average PYLL was 10.4 years (Table 4).

**Table 4:** Absolute number (n), percentage (%), age-standardized mortality rate (ASMR), and potential years of life lost (PYLL) distribution for premature deaths (30–69 years) of Ribeirão Preto residents due to diabetes mellitus, 2010 to 2014, according to sex and year. Ribeirão Preto, state of São Paulo, Brazil, 2017.

Year	Male sex						Female sex						Total					
	Premature death			PYLL			Premature death			PYLL			Premature death			PYLL		
	n	%	ASMR (per 100,000 inhabitants)	n	%	Per death	n	%	ASMR (per 100,000 inhabitants)	n	%	Per death	n	%	ASMR (per 100,000 inhabitants)	n	%	Per death
2010	24	4.1	8.5	243	10.2	10.1	16	2.7	4.6	207	8.7	12.9	40	6.9	6.3	450	18.8	11.3
2011	25	4.3	8.9	270	11.3	10.8	15	2.6	4.2	142	5.9	9.5	40	6.9	6.3	412	17.3	10.3
2012	23	3.9	8.2	271	11.4	11.8	22	3.8	6.2	227	9.5	10.3	45	7.7	7.1	498	20.9	11.1
2013	26	4.5	9.2	260	10.9	10	17	2.9	4.9	133	5.6	7.8	43	7.4	6.9	393	16.5	9.1
2014	31	5.3	10.7	349	14.6	11.3	30	5.2	8.7	285	11.9	9.5	61	10.5	9.6	634	26.6	10.4
Total	129	22.1	45.5	1,393	58.4	10.8	100	17.2	28.6	994	41.6	9.9	229	39.3	36.2	2,387	100	10.4

Source: Division of Epidemiological Surveillance of the Municipal Secretariat of Ribeirão Preto.

## DISCUSSION

The municipality saw a total of 583 DM deaths during the studied period, and an increase in ASMR from 15.6 per 100,000 inhabitants in 2010 to 21.6 in 2014. The Global Burden of Disease study carried out in 195 countries showed that there was an increase in DM mortality in Brazil from 1990 to 2015, as well as an increase in ASMR – from 35.9 per 100,000 inhabitants in 1990 to 37.5 in 2015^16^. Despite the mortality rate increase shown here, our results for Ribeirão Preto (21.6 per 100,000 inhabitants in 2014) are still below the national average. In contrast, a Canadian study showed an approximately one-quarter decline in age-standardized DM mortality between 1995 and 2005^17^.

This study found that the main specific causes of death due to DM were renal complications, responsible for 34.5% of deaths, peripheral circulatory complications, 11.7%, and multiple complications, 8.9%. For 39.1% of deaths, no complications were reported. A Brazilian study evaluating DM mortality between 1996 and 2011, including 294,203 deaths, showed that renal complications (19.1%) and peripheral circulatory complications (6.1%) were the main specific causes of death from DM. It is noteworthy that 7% of the deaths were due to unspecified complications. In the majority (51.9%) of DM deaths in the period, there were no complications^18^.

Acute complications, ketoacidosis and coma accounted for only 1.7% of deaths, which is consistent with the literature^19^, and confirms that the free supply of insulin and other necessities to people with DM was a milestone of the Brazilian health system^19^. When analyzing the obtained results, the higher DM mortality in the female sex (55.1% of deaths) stood out. This percentage was similar to the one found by a Brazilian nationwide study (57.7%) which described DM mortality from 1980 to 2012^4^.

From 2010 to 2014, there was a yearly average ASMR increase of 7% (from 15.5 to 21.6 per 100,000 inhabitants), affecting both sexes. In the same period, the average increase for the male sex was 4% per year (from 17.5 to 21.8 per 100,000 inhabitants), while for the female sex it was 8% per year (from 14.1 to 20.9 per 100,000 inhabitants). The results found here are in agreement with another Brazilian, ecological and time-series study, which evaluated the pattern of DM mortality according to sex from 1980 to 2012^4^. However, despite the fact that in the aforementioned study ASMR also increased for both sexes, the increase rates were lower: 2.9% per year for men (20.8 per 100,000 inhabitants in 1980 to 47.6 in 2012) and 1.7% per year for women (28.7 per 100,000 inhabitants in 1980 to 47.2 in 2012). The study also showed that towards the end of the 1980–2012 period this relationship was reversed, with female mortality higher in the initial years of the survey and male mortality higher in its final years^4^. The ASMR distribution according to sex found by this study showed higher values for men than for women, in all the evaluated years. However, between 2010 and 2014 the yearly increase in average female DM mortality was double that of men, which points to a possible reversal in the future, with higher DM ASMR in the female sex.

ASMR rates increased gradually with age, for both sexes, indicating a dependence on population aging. Among deaths in the female sex, 132 (22.7%) were in the 80 years or more age group, more than double the number of male deaths in the same age group (62, 10.6%). According to estimates by the International Diabetes Federation (2015), women had a greater incidence and prevalence of DM at the apex of their aging when compared to males with the same age and, therefore, a higher mortality rate in the higher age groups^3^. This may be explained by the higher number of premature deaths attributed to external causes in males, a preeminent feature of developing countries, leading to greater survival rates in females, as observed in other studies^3^ ^,^ ^16^ ^,^ ^20^ ^–^ ^23^. A study evaluated 264 causes of death, including DM, in 195 countries from 1980 to 2016, and found that DM deaths in older people over 70 years of age increased by approximately 90% throughout the studied period^24^.

We found an average PYLL loss of 10 years for each premature death (at 30 to 69 years of age), that is, DM withdrew 10 years of economically active life from each person who died from the disease. Therefore, premature deaths together with PYLL strengthen the impact of DM on individuals, families, communities and the country, as it overburdens health services and causes early retirement, harming economic development^3^.

Our study also found an increase in premature mortality rates. The average increase was 5% per year for men (8.5 to 10.7 per 100,000 inhabitants), 14% for women (4.6 to 8.7 per 100,000 inhabitants) and 9% for both sexes (6.3 to 9.6 per 100,000 inhabitants). However, a national study showed a decline in the rate of premature DM mortality in Brazil, which went from 40.6 per 100,000 inhabitants in 2000 to 33.7 in 2011 (a 1.7% yearly reduction). The study projected a drop to 26.9 per 100,000 inhabitants by 2022^14^.

The goal of the national plan for tackling CNCDs is to reduce premature mortality rate to 2% a year^7^, which has not yet been achieved. Thus, although the municipality of Ribeirão Preto, had a marked ASMR increase in both premature and overall deaths – and mainly in the female sex – local death rates were still lower than the national averages. These discrepancies suggest that a general increase in the incidence and prevalence of DM is occurring. A plausible explanation for this situation is the disorganized industrial and urban growth of most Brazilian municipalities, coupled with the adoption of harmful life habits by the majority of the population, which increase the likelihood of developing chronic diseases such as DM^25^.

The deleterious long-term effects of hyperglycemia on people suffering from DM, associated with the increase in premature mortality, calls for reflection on public health policies, in order to propose effective interventions against DM. Thus, this study may contribute to advances in local health diagnosis, and serve as basis for new subsidies towards preventive and health promotion measures, with a consequent reduction in the rates of premature mortality and DM mortality^26^.

Another study described mortality rates in all Brazilian capitals, from 1980 to 2007^27^, and observed increases in ASMR mainly in North and Northeast capitals. The city of Porto Velho, state of Rondônia, had an ASMR of 9.76 per 100,000 inhabitants in the first four years, and this number increased to 46.13 in the last four years. In São Luís, state of Maranhão, ASMR increased from 24.75 in the first quadrennium to 54.38 in the last.

In the Southeastern region, two capitals had a decline in ASMR: Belo Horizonte, state of Minas Gerais, and São Paulo, state of São Paulo, with a drop from 28.82 per 100,000 inhabitants in the first four-year period to 18.36 in the last, and from 28.94 in the first four-year period to 22.05 in the last, respectively^27^. These data, however, differs from the results of our study, which, although performed in the same geographic region, showed an increase in ASMR during the evaluated period.

Accessibility to health services may be a determining factor of such macro-regional contrasts. In fact, a nationwide study^28^ on the population's access to primary care teams certified by the National Program for Access and Quality Improvement in Primary Care (PMAQ-AB) found a greater possibility of access in the Southeast, a lesser possibility in the North, and a greater concentration of family health teams in the capitals^28^. These data highlight the relevance of studies such as ours and the need for future research analyzing DM mortality rates and other CNCDs in medium or small municipalities.

One of this study's limitations was the underreporting of the disease in death certificates, which may have led to an underestimation of DM mortality rates. Also, there was no investigation and redistribution of possible insufficiently defined causes of death related to endocrine diseases, as performed in other studies^14^ ^,^ ^18^ ^,^ ^29^. Another limitation of this study is the impossibility of describing mortality according to specific DM types, since the majority of the deaths were classified as E14 (84.7%)^19^. In order to reduce underreporting, it is necessary to reinforce the importance of adequate completion of the death certificate by the physician. Furthermore, epidemiological surveillance teams trained in monitoring all spheres have to be implemented, in order to investigate insufficiently defined causes^30^.

In conclusion, the results show that DM mortality in the city of Ribeirão Preto, state of São Paulo, Brazil increased in the 2010 to 2014 period. There was a higher occurrence of deaths in females, but ASMRs were higher in males. As to age, there was a predominance of deaths in the 80 years or more age group. For both sexes, there was an annual mean increase of 9% in premature mortality during the studied period. DM deaths are responsible for the withdrawal of 10 years of life expectancy. These results may support public policies in the prevention of DM mortality, as well as health promotion towards adult individuals with DM.
